# Cultivation of Corneal Endothelial Cells on a Pericellular Matrix Prepared from Human Decidua-Derived Mesenchymal Cells

**DOI:** 10.1371/journal.pone.0088169

**Published:** 2014-02-05

**Authors:** Ryohei Numata, Naoki Okumura, Makiko Nakahara, Morio Ueno, Shigeru Kinoshita, Daisuke Kanematsu, Yonehiro Kanemura, Yoshiki Sasai, Noriko Koizumi

**Affiliations:** 1 Department of Biomedical Engineering, Faculty of Life and Medical Sciences, Doshisha University, Kyotanabe, Japan; 2 Department of Ophthalmology, Kyoto Prefectural University of Medicine, Kyoto, Japan; 3 Division of Regenerative Medicine, Institute for Clinical Research, Osaka National Hospital, National Hospital Organization, Osaka, Japan; 4 RIKEN Center for Developmental Biology, Hyogo, Japan; University of Reading, United Kingdom

## Abstract

The barrier and pump functions of the corneal endothelium are essential for the maintenance of corneal transparency. Although corneal transplantation is the only current therapy for treating corneal endothelial dysfunction, the potential of tissue-engineering techniques to provide highly efficient and less invasive therapy in comparison to corneal transplantation has been highly anticipated. However, culturing human corneal endothelial cells (HCECs) is technically difficult, and there is no established culture protocol. The aim of this study was to investigate the feasibility of using a pericellular matrix prepared from human decidua-derived mesenchymal cells (PCM-DM) as an animal-free substrate for HCEC culture for future clinical applications. PCM-DM enhanced the adhesion of monkey CECs (MCECs) via integrin, promoted cell proliferation, and suppressed apoptosis. The HCECs cultured on the PCM-DM showed a hexagonal morphology and a staining profile characteristic of Na^+^/K^+^-ATPase and ZO-1 at the plasma membrane in vivo, whereas the control HCECs showed a fibroblastic phenotype. The cell density of the cultured HCECs on the PCM-DM was significantly higher than that of the control cells. These results indicate that PCM-DM provides a feasible xeno-free matrix substrate and that it offers a viable in vitro expansion protocol for HCECs while maintaining cellular functions for use as a subsequent clinical intervention for tissue-engineered based therapy of corneal endothelial dysfunction.

## Introduction

The corneal endothelium is the inner layer of the cornea, and it plays an essential role in the maintenance of corneal transparency via its barrier and pump functions [1]. A distinct feature of human corneal endothelial cells (HCECs) in the clinical setting is that they are essentially nonregenerative in vivo [2]. Severe damage of CECs due to Fuchs' corneal endothelial dystrophy, trauma, or surgical intervention causes corneal blindness associated with decompensation of the barrier and pump functions of the corneal endothelium [2]. Corneal transplantation is the only treatment option, and no pharmaceutical treatment is available [3]. Although less invasive corneal transplantation techniques, such as Descemet's stripping automated endothelial keratoplasty (DSAEK) and Descemet's membrane endothelial keratoplasty (DMEK), have been developed and have become very popular [4], [5], there are still transplantation-associated problems [6]. For instance, there is a severe worldwide shortage of donor corneas, 20% of grafts are rejected after 5 years, and transplanted corneal endothelium is subject to continual loss of cell density [3], [7]. Tissue-engineering techniques have been strongly anticipated to overcome these problems and to provide highly efficient therapy [3]. Researchers have used tissue engineering-based techniques to transplant cultured CECs in animal corneal endothelial dysfunction models and to resolve corneal transparency [8]–[13]. Coincident to other organs, such as heart [14], pancreas [15], cartilage [16], and corneal epithelium [17], regenerative therapy for corneal endothelium is expected to be introduced in clinical settings.

The critical technical difficulty that must be overcome before tissue engineering therapy of corneal endothelium can be introduced in clinical settings is the in vitro expansion of HCECs [18]. Although HCECs are cultured in several laboratories, there is no established protocol, especially for clinical use [18]. Any protocol must overcome the following important obstacles: HCECs exhibit massive apoptosis during isolation from donor cornea [19], they undergo endothelial-mesenchymal transformation with loss of cellular functions [20], and they display potent limited proliferative ability even in vitro [21], [22]. One important approach to culture HCECs is the use of extracellular matrix (ECM) as the culture substrate. For instance, ECM derived from bovine CECs [23] and FNC Coating Mix^®^ (Athena Environmental Sciences) [24] were used for HCEC culture. However, these are animal-derived matrixes and raise the possibility of contamination with xenogenic pathogens and immunogens. Accordingly, to expand HCECs for clinical applications, it is desirable to minimize animal-derived ECM in the culture to diminish the risk of infections caused by animal-origin pathogens.

Human pluripotent cells, such as ES and iPS cells, are routinely derivated and maintenance cultured and are anticipated as a cellular source for tissue engineering. Matrigel derived from a mouse EHS sarcoma cell line has been commonly used for maintenance culture of human ES cells and iPS cells [25]. The maintenance-supporting potency of several matrixes to accomplish a xeno-free cultivation procedure for clinical use of human pluripotent cells has been studied [26]. A pericellular matrix of decidua-derived mesenchymal cells (PCM-DM) was reported to be a highly potent culture substrate for human ES cells [26] and human iPS cells [27]. As decidua-derived mesenchymal cells (DMCs) are isolated from human fetal membrane (FM) [28], [29], PCM-DM offers a human-derived xeno-free culture-supporting matrix.

In the present study, we showed that PCM-DM is a potent substrate for an in vitro expansion culture protocol of HCECs. We demonstrated that PCM-DM enhances cell adhesion via integrin, promotes cell proliferation, and suppresses apoptosis. These results indicate that PCM-DM provides a feasible xeno-free matrix substrate and a substantial in vitro expansion protocol for HCECs while maintaining cellular functions for use as a subsequent clinical intervention for tissue-engineered based therapy of corneal endothelial dysfunction.

## Materials and Methods

### Ethics statement

The human tissue used in this study was handled in accordance with the tenets set forth in the Declaration of Helsinki. Informed written consent was obtained from the next of kin of all deceased donors in regard to eye donation for research. Human full-term FMs were collected from normal healthy mothers at the Osaka National Hospital with their written informed consent [29]. Approval to use the human FMs was obtained from the ethical committees of both the Osaka National Hospital and RIKEN CDB and Doshisha University. Human donor corneas were obtained from SightLife^TM^ (http://www.sightlife.org/, Seattle, WA). All tissues were recovered under the tenets of the Uniform Anatomical Gift Act (UAGA) of the particular state in which the donor consent was obtained and the tissue was recovered. The monkey corneas used in this study were handled in accordance with the ARVO Statement for the Use of Animals in Ophthalmic and Vision Research. The general welfare of the animals from which the corneas were harvested and isolation of the tissue were approved by the institutional animal care and use committee of Nissei Bilis Co., Ltd. (Otsu, Japan). Nissei Bilis Co., Ltd. s a contract research organization evaluated as “grade A” facility by the Good Laboratory Practice (GLP) inspection. Nissei Bilis Co., Ltd. was also accredited by the Center for the Accreditation of Laboratory Animal Care and Use set up in the Japan Health Sciences Foundation. Institutional Animal Care and Use Committee was set up in Nissei Bilis Co., Ltd. and all procedures for animal care and experimental procedures were performed according to the standard operating procedure with intensive consideration for animal rights and welfare. The cynomolgus monkeys were housed in individual stainless steel cages equipped with perch and monkey bars at Nissei Bilis Co., Ltd. As environment enrichment, Wood Enrichment Products (Bio-Serve, Frenchtown, NJ), Kong Toys on Chain, Red, Certified (Bio-Serve), and Primate Busy Box (Bio-Serve) were provided. Each cage was provided with reverse-osmosis water delivered by an automatic water supply system and supplied with experimental animal diet (PS-A; Oriental Yeast Co., Ltd., Tokyo, Japan). The room temperature was controlled by heating units inside the rooms and was maintained at 18.0–26.0°C. The humidity was maintained at 29.5 to 80.4%. The animals were maintained on a 12:12-h light:dark cycle (lights on 7 a.m. to 7 p.m.). The corneas used in the present study were obtained from animals euthanized with an overdose of intravenous pentobarbital sodium according to the guidelines on euthanasia of American Veterinary Medical Association (AVMA) for other research purposes. The corneas of cynomolgus monkeys were harvested after confirmation of cardiopulmonary arrest by veterinarians and then provided by Nissei Bilis Co., Ltd. for our research.

### Preparation of pericellular matrix of decidua-derived mesenchymal cells (PCM-DM)

The DMCs were cultivated using a protocol described previously [29]. Briefly, the human decidual tissues were mechanically isolated from the FMs. They were then dissected into small pieces and enzymatically dissociated by 1 mg/mL collagenase A (Life Technologies, Carlsbad, CA), 1 mg/mL dispase (Life Technologies), and 0.01% DNase I (1 mg/mL, Life Technologies). The DMCs were then filtered and seeded at a density of 2×10^4^ cells/mL and cultured with DMEM/F-12 (1:1)-based culture medium (Life Technologies), supplemented with 10% fetal bovine serum (FBS), 15 mM HEPES (Life Technologies) and antibiotic-antimycotic (Life Technologies) at 37°C in 5% CO_2_. The culture medium was replaced twice a week, and the DMCs were passaged using 0.05% trypsin-EDTA (Life Technologies) once a week. The PCM-DM was prepared by a previously reported method [26], [27]. Briefly, the DMCs were plated at a density of 3.5 ×10^4^ cells/cm^2^ onto a culture plate coated with 0.1% gelatin and cultured for 3 days after reaching confluence with the DMEM/F-12 (1:1)-based culture medium. After rinsing with phosphate-buffered saline (PBS), the DMCs were lysed at 4°C for 30 min with deoxycholate solution (0.5% sodium deoxycholate in 10 mM Tris-HCl, pH 8.0). After DMC lysis, the dishes were washed six times with PBS by pipetting to flush off the cell debris. The treated dishes were stored under semidry conditions at 4°C.

### Cell culture of monkey CECs (MCECs)

Eight corneas from four cynomolgus monkeys (3–5 years-of-age; estimated equivalent human age: 5–20 years) housed at Nissei Bilis Co., Ltd. were used for the monkey CEC (MCEC) culture. The MCECs were cultivated using a modified protocol described previously [12]. Briefly, the Descemet's membrane, including the CECs, was stripped and digested at 37°C for 2 h with 1 mg/mL collagenase A. The MCECs were then seeded on a culture plate coated with FNC Coating Mix^®^ (Athena Environmental Sciences) and cultured with growth medium composed of Dulbecco's modified Eagle's medium (Life Technologies), supplemented with 10% FBS, 50 U/mL penicillin, 50 µg/mL streptomycin, and 2 ng/mL FGF-2 (Life Technologies). The MCECs were then cultured in a humidified atmosphere at 37°C in 5% CO_2_, and the culture medium was changed every 2 days. When the MCECs reached confluence, they were trypsinized with 0.05% Trypsin-EDTA (Life Technologies) and passaged at ratios of 1:2–4. MCECs after the 5–9th passage were used for these experiments.

### Cell culture of human CECs (HCECs)

Four human donor corneas (>60 years-of-age) were used for the HCECs culture. The HCECs were cultured according to modified published protocols [20], [24]. Briefly, the Descemet's membrane, including the CECs, was stripped and digested at 37°C for 12 h with 1 mg/mL collagenase A. After digestion at 37°C, the HCECs obtained from individual corneas were seeded in one well of a 12-well plate coated with FNC Coating Mix^®^ (Athena Environmental Sciences). The culture medium was prepared according to published protocols. Briefly, basal medium containing OptiMEM-I (Life Technologies), 8% FBS, 5 ng/mL epidermal growth factor (Sigma-Aldrich Co., St. Louis, MO), 20 µg/mL ascorbic acid (Sigma-Aldrich), 200 mg/L calcium chloride (Sigma-Aldrich), 0.08% chondroitin sulfate (Wako Pure Chemical Industries, Ltd., Osaka, Japan), and 50 µg/mL of gentamicin was prepared, and the conditioned medium was then recovered as culture medium after cultivation of inactivated 3T3 fibroblasts. Inactivation of the 3T3 fibroblasts was performed as described previously [20], [30]. The HCECs were cultured in a humidified atmosphere at 37°C in 5% CO_2_, and the culture medium was changed every 2 days. When the HCECs reached confluence in 14–to 28 days, they were rinsed in Ca^2+^ and Mg^2+^-free PBS, trypsinized with 0.05% Trypsin-EDTA for 5 min at 37°C, and passaged at ratios of 1:2. To evaluate the effect of extracellular matrixes on cell density, the HCECs were seeded on collagen 1 (BD Biosciences, Franklin Lakes, NJ), collagen 4 (BD Biosciences ), fibronectin (BD Biosciences), or a PCM-DM coated dish.

### Immunocytochemistry

To evaluate the PCM-DM, the DMCs were plated at a density of 3.5×10^4^ cells/cm^2^ onto Corning^®^ Transwell^®^ inserts (0.4 µm pore) coated with 0.1% gelatin and cultured for 3 days after reaching confluence, and then lysed with deoxycholate solution. The whole membrane of the Corning^®^ Transwell^®^ was fixed with 2.5% glutaldehyde and 2% paraformaldehyde in 0.1 M sorensen phosphate buffer for 2 h. The membrane was embedded into Tissue-Tek^®^ O.C.T. Comound (Sakura Finetek Co., Ltd., Tokyo, Japan), and sections (14 µm) were obtained. The cultured MCECs or HCECs seeded into Lab-Tek^TM^ Chamber SlidesT^M^ (NUNC A/S, Roskilde, Denmark) coated with or without PCM-DM were fixed in 4% paraformaldehyde for 10 min at room temperature and incubated for 60 min with 1% bovine serum albumin (BSA). Actin staining was performed with a 1:400 dilution of Alexa Fluor^®^ 488-conjugated phalloidin (Life Technologies). The samples were incubated with a 1:200 dilution of fibronectin (BD Biosciences), collagen 4 (Abcam, Cambridge, UK), ZO-1 (Zymed Laboratories, South San Francisco, CA) polyclonal antibody, and a 1:200 dilution of Na^+^/K^+^-ATPase (Upstate Biotec, Lake Placid, NY) monoclonal antibody for 1 h at room temperature. After washing with PBS, Either Alexa Fluor^®^ 488-conjugated (Life Technologies) or Alexa Fluor^®^ 594-conjugated goat antimouse IgG (Life Technologies) was used for the secondary antibody with a 1:2000 dilution. Nuclei were stained with DAPI (Vector Laboratories, Burlingame, CA). The slides were then inspected by fluorescence microscopy (TCS SP2 AOBS; Leica Microsystems, Welzlar, Germany).

### Cell adhesion assay

The MCECs were seeded in 48-well or 96-well plates coated with or without PCM-DM. After 24 h, the mean cell areas were evaluated by Image J software (U.S. National Institutes of Health, Bethesda, MD) following actin staining. The numbers of adhered cells after 1 h of seeding were measured with a CellTiter-Glo^TM^ luminescent cell viability assay (Promega Corporation, Madison, WI) according to the manufacturer's instructions. The number of adhered MCECs at 1 h after seeding was then measured using a Veritas^TM^ microplate luminometer (Promega Corporation). To elucidate the effect of the interaction between the integrins of the CECs and the PCM-DM on cell adhesion, the MCECs (1×10^4^ cells/well) were seeded in 96-well plates coated with PCM-DM in the presence or absence of integrin-neutralizing antibodies (2 µg/mL), anti-α_1_ integrin (Merck Millipore, Billerica, MA), anti-α_v_ integrin (Merck Millipore), and anti-β_1_ integrin (R&D systems Inc., Minneapolis, MN). After 3 h of seeding, adherent cells were measured with the CellTiter-Glo^TM^ luminescent cell viability assay.

### Immunoblotting

For immunoblotting, the cells were washed with PBS and then lysed with a radio immunoprecipitation assay buffer (Bio-Rad Laboratories, Hercules, CA) containing a phospatase inhibitor cocktail 2 (Sigma-Aldrich) and protease inhibitor cocktail (Nacalai Tesque, Kyoto, Japan). The lysates were then centrifuged at 15,000 rpm for 10 min at 4°C. The resultant supernatant was collected, and the protein concentration of the sample was assessed with a BCA™ protein assay kit (Pierce Biotechnology Rockford, IL). The proteins were separated by sodium dodecyl sulfate polyacrylamide gel electrophoresis (SDS-PAGE) and transferred to polyvinylidene fluoride membranes. The membranes were blocked with 5% nonfat dry milk (Cell Signaling Technology, Inc., Danvers, MA) in TBS-T buffer and incubated with the following primary antibodies: Akt, phospho-Akt Ser 473, phospho-paxillin Tyr118, FAK, phospho-FAK Thy397 (Cell Signaling Technology), and GAPDH (Medical & Biological Laboratories CO., Ltd., Nagoya, Japan) for 1 h at room temperature. They were also incubated with HRP-conjugated antirabbit or antirabbit IgG secondary antibody (Cell Signaling Technology) (1:5000 dilutions). The membranes were exposed by an ECL Advance Western blotting detection kit (GE Healthcare, Piscataway, NJ) and then examined with the LAS4000S (Fujifilm, Tokyo, Japan) imaging system.

### Cell proliferation assay

Cell proliferation was evaluated by 5-ethynyl-2 Click-iT^®^ EdU imaging kits (Life Technologies) according to the manufacturer's instructions. Briefly, the MCECs (1×10^4^ cells/well) were cultured in a 48-well plate coated with or without PCM-DM and incubated with 10 µM EdU for 24 h at 37°C. The MCECs were fixed with 4% paraformaldehyde for 20 min at room temperature, permeabilized with 0.3% Triton**^®^** X-100 (Nacalai Tesque), and washed with 3% BSA in PBS. The MCECs were then incubated with a reaction cocktail for 30 min at room temperature. The slides were stained with DAPI and examined under a fluorescence microscope (BZ-9000; Keyence, Osaka, Japan). DNA synthesis was also measured by incorporation of 5′-bromo-2-deoxyuridine (BrdU) using the Cell Proliferation Biotrak ELISA system version 2 (Amersham Biosciences, Freiburg, Germany) according to the manufacturer's instructions. Briefly, the MCECs (5×10^3^ cells/well) were cultured in a 96-well plate coated with or without PCM-DM. The MCECs were incubated with 10 µM BrdU for 24 h at 37°C. The cultured cells were incubated with a fixative solution, blocking solution, and monoclonal antibody against BrdU for 90 min. The BrdU absorbance was measured with a spectrophotometric microplate reader at a test wavelength of 450 nm.

### Scratch-induced directional migration assay

The MCECs were cultured in 6-well plates coated with or without PCM-DM. MCECs cultured at confluence for 1 week were scraped with a 1000 µl plastic pipette tip to create six linear defect sites. Wound closure was serially measured for 18 h, and cell migrations were observed by phase-contrast microscopy. The widths of the wound were measured using Image J software. Six defect sites were measured for each experiment.

### TUNEL assay

To elucidate the antiapoptotic effect of PCM-DM, MCECs were cultured in 48-well plates coated with or without PCM-DM. They were incubated for 72 h after removal of serum. The number of apoptotic cells was measured with the DeadEnd^TM^ Fluorometric TUNEL System (Promega Corporation) according to the manufacturer's instructions. Briefly, the MCECs were fixed with 4% paraformaldehyde for 10 min at room temperature and permeabilized with 0.2% Triton**^®^** X-100 in PBS for 5 min at room temperature. They were then equilibrated with equilibration buffer for 10 min at room temperature and incubated with equilibration buffer in a nucleotide mix and rTdT enzyme for 1 h at 37°C. The nuclei were stained with DAPI, and apoptotic cells were detected by fluorescence microscopy (TCS SP2 AOBS; Leica Microsystems).

### Statistical analysis

The statistical significance (*P*-value) of the mean values in the two-sample comparison was determined by use of the Student's *t*-test. The statistical significance in the comparison of multiple sample sets was analyzed by Dunnett's multiple-comparisons test. Values shown on the graphs represent the mean ± SEM.

## Results

### Effect of PCM-DM on cell adhesion

**Figure 1:**
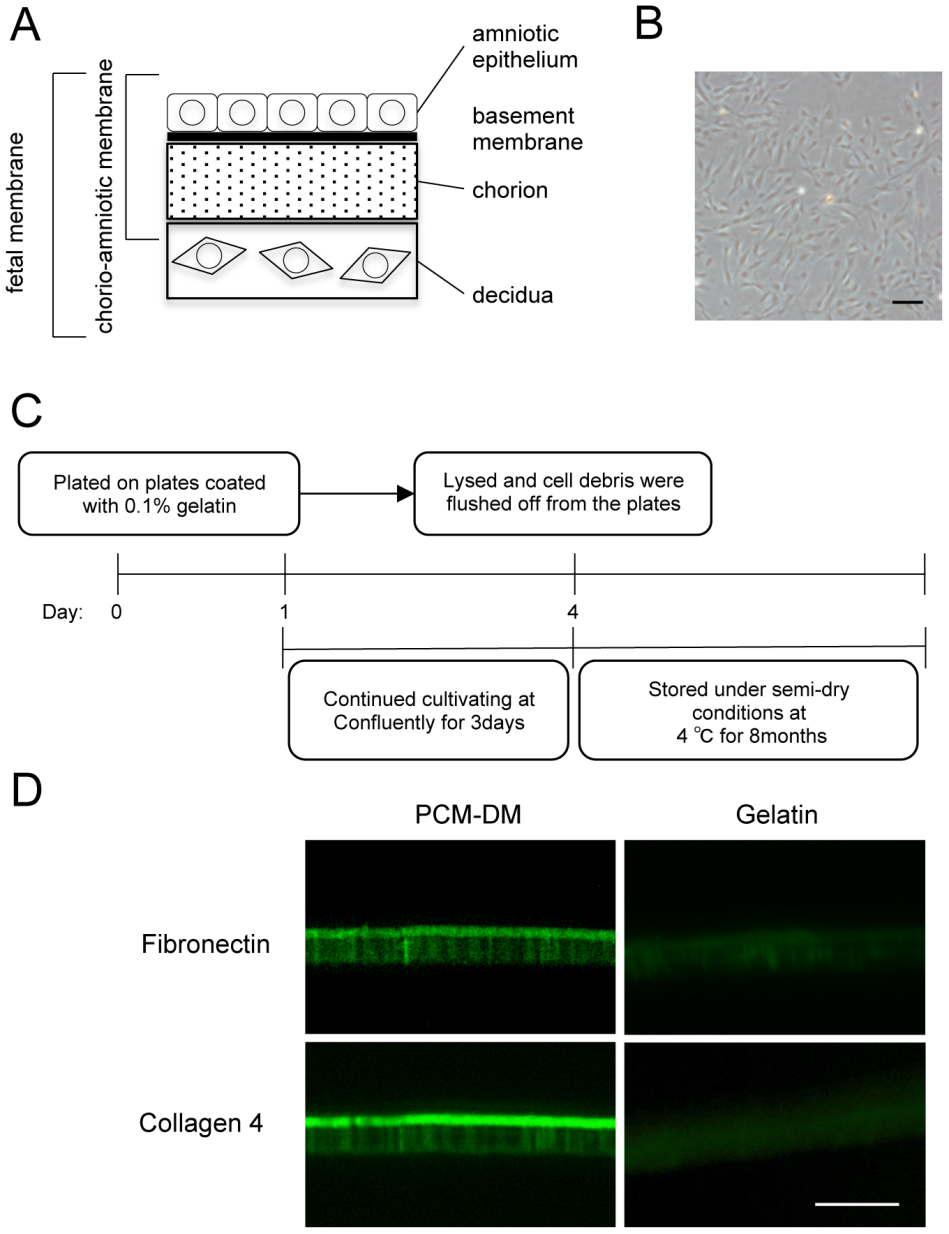
Preparation of pericellular matrix prepared from human decidua-derived mesenchymal cells (PCM-DM) for culture substrate. (A) Schematic of the structure of human fetal membrane (FM). Human FM consists of three main layers: amniotic, chorionic, and decidual membranes. (B) Representative phase-contrast image of human decidua-derived mesenchymal cells (DMCs). The human decidual tissues were isolated from the FMs. The DMCs were isolated from the decidua and cultured with DMEM/F-12 (1:1)-based culture medium supplemented with 10% fetal bovine serum (FBS) (scale bar, 200 µm). (C) The procedure for preparation of the PCM-DM from the DMCs. The DMCs were plated at a density of 3.5×10^4^ cells/cm^2^ onto the culture plate coated with 0.1% gelatin and cultured for 3 days after reaching confluence. They were then lysed with deoxycholate solution (0.5% sodium deoxycholate in 10 mM Tris-HCl, pH 8.0). PCM-DM coated dishes stored under semidry conditions at 4°C for less than 8 months were used for the experiments. (D) The DMCs were plated at a density of 3.5×10^4^ cells/cm^2^ onto Corning^®^ Transwell^®^ inserts coated with 0.1% gelatin and cultured for 3 days after reaching confluence, and then lysed with deoxycholate solution. PCM-DM was evaluated by fibronectin and collagen 4 staining (scale bar, 50 µm).

**Figure 2:**
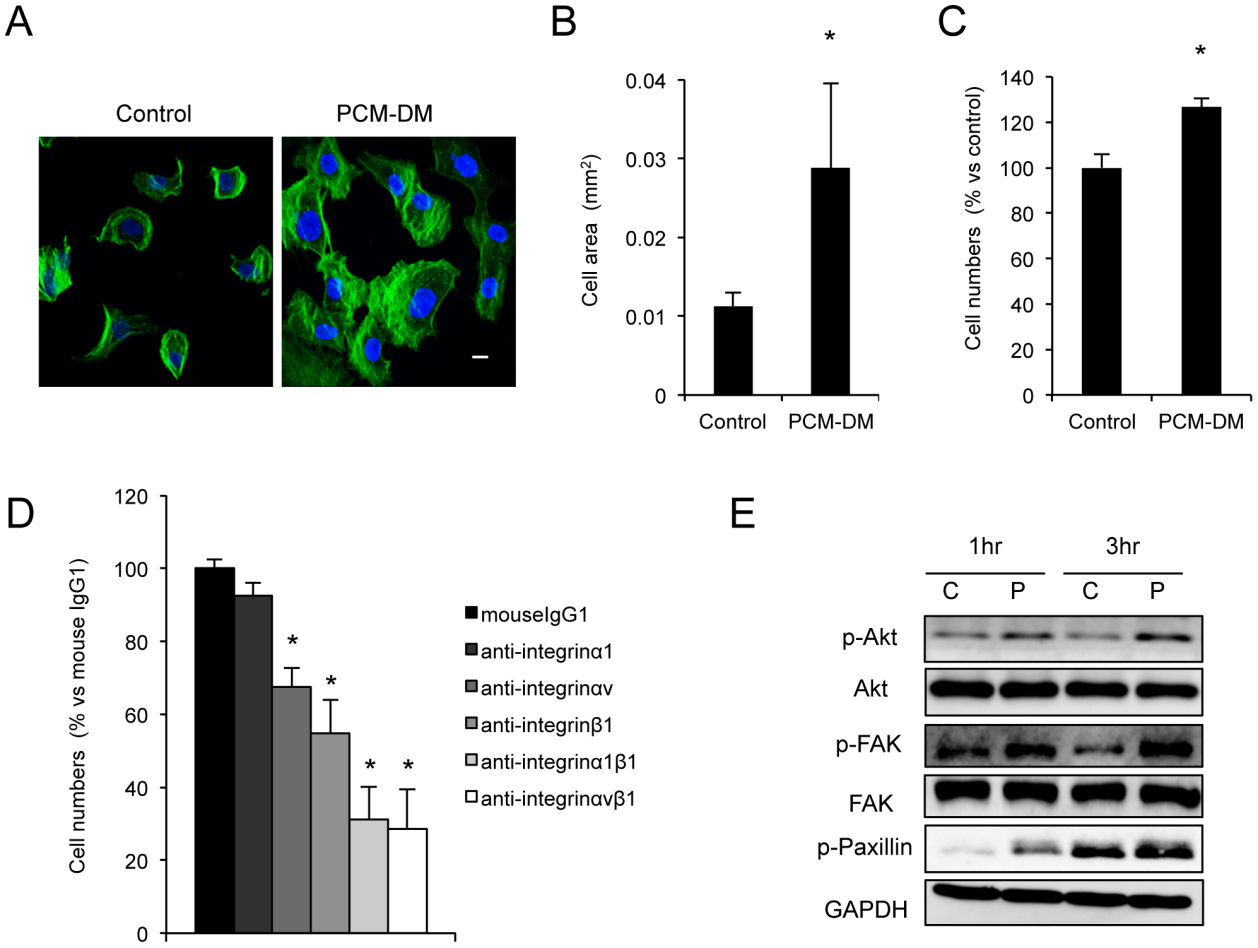
Effect of PCM-DM on cell adhesion. (A+B) Monkey CECs (MCECs) were seeded at a density of 2.5×10^3^ cells/well, and their cell morphology was evaluated by actin immunostaining after 24 h. The cell area was measured by Image J software (National Institutes of Health), and the mean cell area was plotted (*n* = 10). The pictures are representative of two independent experiments *p<0.01; scale bar, 100 µm. (C) The MCECs were seeded at a density of 5.0×10^3^ cells/well, and the number of adhered MCECs was examined with a CellTiter-Glo^TM^ luminescent cell viability assay after 1 h. The experiments were performed in triplicate. *p<0.01. (D) The MCECs were seeded at a density of 1.0×10^4^ cells/well and incubated with DMEM supplemented with 10% FBS with 2 µg/mL neutralizing antibodies (α1, αv, and/or β1 integrin subunits). The number of adhered MCECs was examined with a CellTiter-Glo^TM^ luminescent cell viability assay after 3 h. The experiments were performed in triplicate. *p<0.01. (E) MCECs seeded on the PCM-DM or the control culture dish for 1 or 3 h. Phosphorylation of Akt, FAK, and paxillin was evaluated by Western blot analysis. The experiments were performed in duplicate.

As MCECs exhibit limited proliferative ability, an in vitro expansion procedure for these cells has not been established. Human decidual tissues were isolated from the FMs, and DMCs were isolated from the decidua and cultured (Fig. 1A, B). To coat the culture dish with PCM-DM, the DMCs were cultured for 3 days at confluence and lysed (Fig. 1A, C). As PCM-DM is mainly composed of collagen IV and fibronectin [26], we confirmed the positive expression of those extracellular matrices by immunostaining (Fig. 1D). Then, we examined the effect of the PCM-DM on the adhesion of the CECs onto the substrate. As primate CECs have limited proliferative ability unlike rodent CECs [12], [20], [31], [32], we used MCECs as a substitute for HCECs. The MCECs cultured on the PCM-DM showed lamellipodia and stress fibers, whereas the control MCECs cultured on the noncoated dish exhibited less formation of lamellipodia and stress fibers (Fig. 2A). The PCM-DM significantly enhanced the cell area in comparison to the control plate (0.029 µmm^2^ and 0.011 µmm^2^, respectively) after 24 h of seeding (Fig. 2B). The number of adhered cells at 1 h showed a 1.2-fold increase when the MCECs were seeded on the PCM-DM (Fig. 2C). The involvement of integrins, which are transmembrane receptors composed of two chains (α and β units) [33], in the adhesion of the MCECs was evaluated. The α1, αv, and/or β1 integrin subunits are known to recognize collagen IV and fibronectin as a ligand. Using neutralizing antibodies of α1, αv, and/or β1 integrin subunits, we revealed that inhibition of these subsets, except α1, significantly downregulated the adhesion of the MCECs on the PCM-DM (Fig. 2D). In addition, phosphorylation of focal adhesion-related proteins, such as Akt, FAK, and paxillin, was enhanced in the cells cultured on the PCM-DM in comparison to the control cells (Fig. 2E).

### Effect of PCM-DM on cell proliferation and wound healing

**Figure 3:**
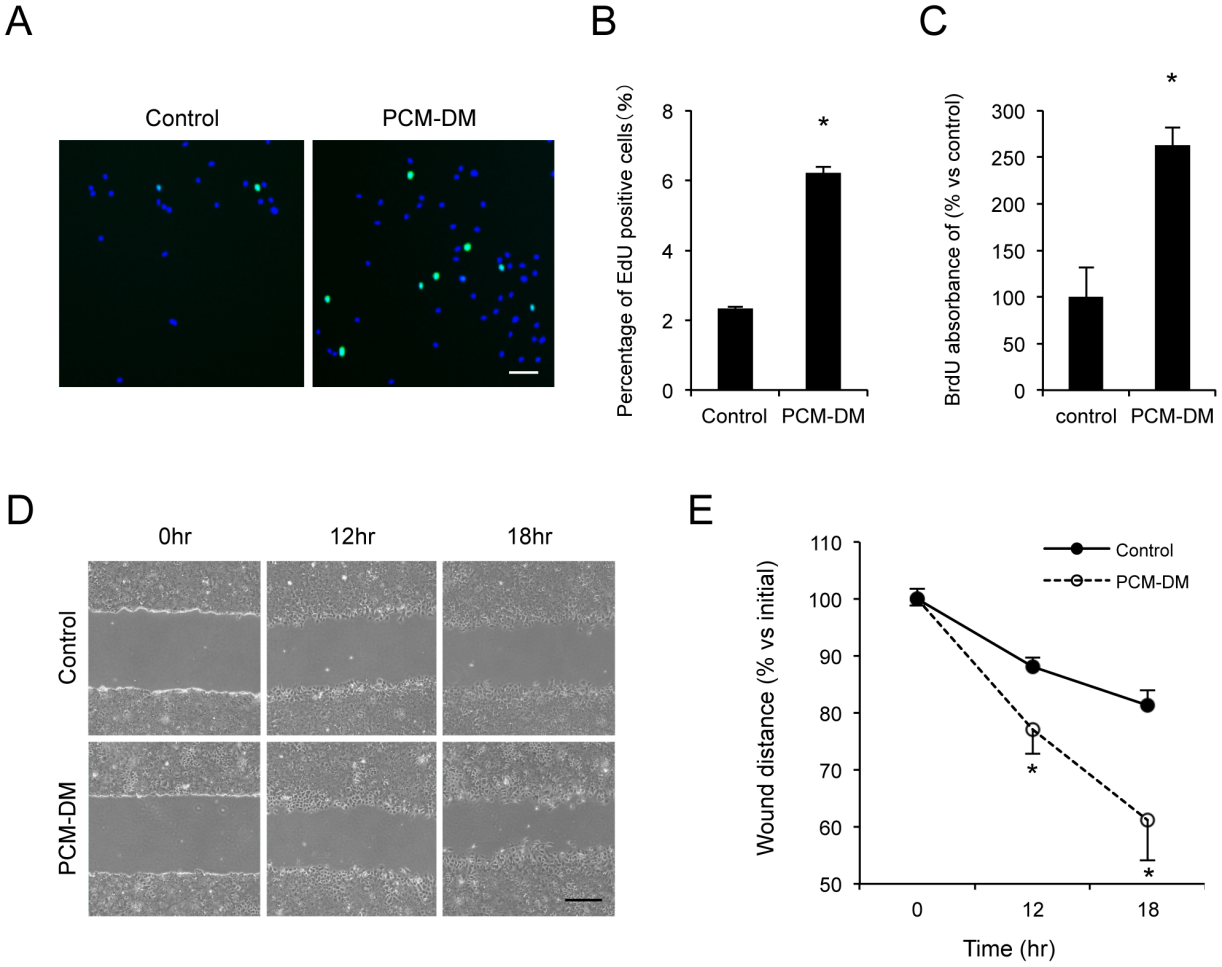
Effect of PCM-DM on the promotion of cell proliferation and migration. (A+B) To test the proliferative potential, the MCECs cultured on the PCM-DM were immunostained with 5-ethynyl-2 Click-iT^®^ EdU Imaging Kits, and the percentages of EdU-positive cells were evaluated after 24 h of incubating with 10 µM EdU. The experiments were performed in duplicate. Scale bar: 100 µm; *p<0.01. (C) The MCECs were seeded at a density of 5.0×10^3^ cells/well. The proliferation of the MCECs was evaluated by a BrdU incorporation assay after 24 h of incubating with 10 µM BrdU. The experiment was performed in duplicate. *p<0.01. (D+E) The MCECs were cultured for 14 days after reaching confluence, and the monolayer cells were wounded by scratching. After 12 and 18 h, the wound distance was quantified by Image J software (*n* = 10). The experiment was performed in triplicated. *p<0.01; scale bar: 200 µm.

We next determined the effect of the PCM-DM on the cell proliferation of the MCECs. The newly synthesized DNA of the MCECs cultured on the PCM-DM was immunostained with 5-ethynyl-2 Click-iT^®^ EdU imaging kits (Fig. 3A). The control cells cultured on the noncoated dish showed 2.3% EdU-positive cells, whereas the MCECs cultured on the PCM-DM showed 6.2% EdU-positive cells (Fig. 3B). BrdU ELISA also showed that incorporation of BrdU into the newly synthesized DNA was increased 2.6-fold in the MCECs on the PCM-DM compared to the control cells (Fig. 3C). A scratch-induced directional migration assay was employed to compare the wound closure of the MCECs cultured on the PCM-DM to that of the control cells. The wound was created in the cultured confluent MCECs, and the wound closure was measured 18 h after the initial wounding (Fig. 3D). The wound distance of the MCECs cultured on the PCM-DM decreased 61% of the initial wound distance, whereas it deceased 81% in the control MCECs (Fig. 3E).

### Effect of PCM-DM on apoptosis

**Figure 4:**
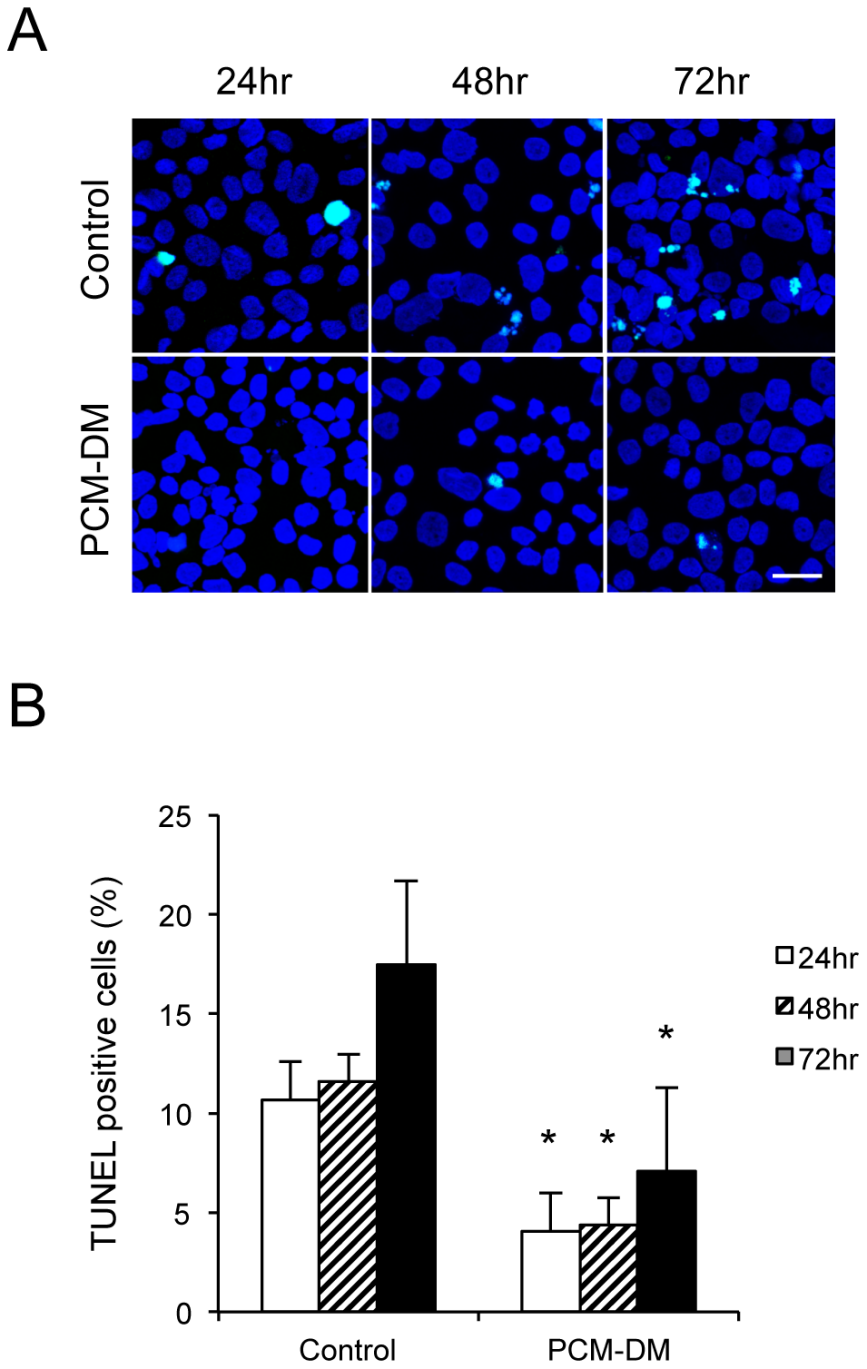
Antiapoptotic effect of PCM-DM. (A) To evaluate the effect of PCM-DM on the apoptosis of MCECs, serum was removed from the culture medium to induce apoptosis. TUNEL staining was performed to evaluate DNA fragmentation during apoptosis after 24, 48, and 72 h. Scale bar: 50 µm. (B) The percentages of TUNEL-positive apoptotic cells were evaluated (*n* = 3). The experiment was performed in duplicate. *p<0.01.

Regulating apoptosis in an in vitro culture is vital because CECs tend to exhibit apoptosis primarily during culture or passage. TUNEL staining was used to evaluate the effect of the PCM-DM on the apoptosis of the MCECs. Serum was removed from the culture medium for 72 h to induce apoptosis, and TUNEL staining was performed to evaluate DNA fragmentation during apoptosis (Fig. 4A). The percentages of TUNEL-positive apoptotic cells were 7.1% among the MCECs cultured on the PCM-DM and 17.5% among control MCECs cultured on the noncoated dish after 72 h of serum removal (Fig. 4B).

### Phenotype of human corneal endothelial cells (HCECs) on PCM-DM

**Figure 5:**
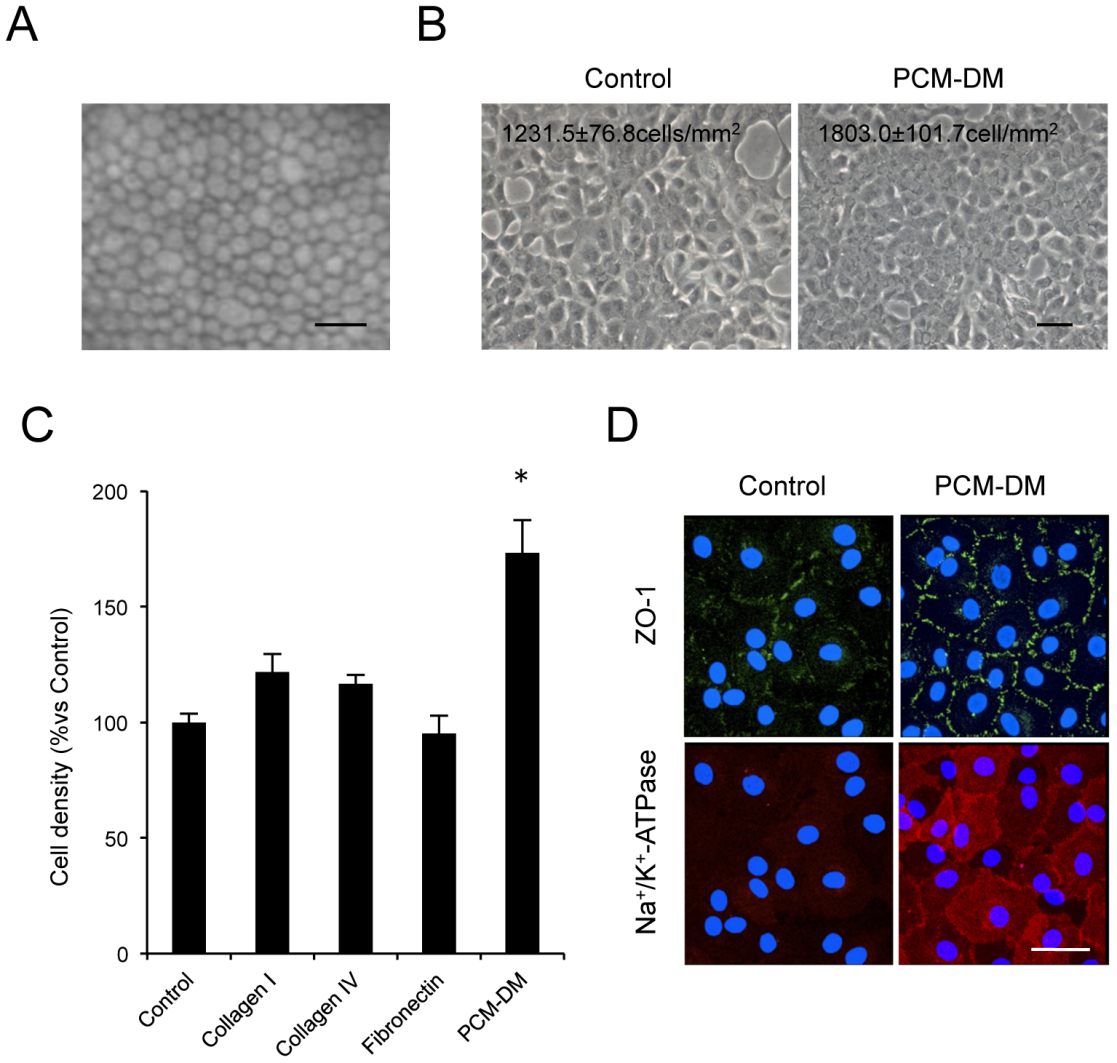
Characteristics of cultivated HCECs on PCM-DM. (A) An image of corneal endothelium of a normal human subject was obtained by in vivo contact specular microscopy. Scale bar: 100 µm. (B) The HCECs (passage 3) were passaged on the PCM-DM and on the control culture dish and cultured for 30 days to form a monolayer sheet. Representative phase contrast images are shown. Scale bar 100 µm. The experiments were performed in triplicate. (C) The HCECs were passaged on the noncoated plate (control), collagen 1, collagen 4, fibronectin, and PCM-DM and cultured for 30 days. The cell densities of the HCECs were evaluated by KSS-400EB software. The experiments were performed in triplicate. (D) ZO-1 and Na^+^/K^+^-ATPase at the plasma membrane was stained in the HCEC culture on the PCM-DM and on the control culture plate. Scale bar 50 µm.

The findings that PCM-DM enabled integrin-dependent cell adhesion to the substrate, enhanced cell proliferation, and suppressed apoptosis in MCECs led us to examine whether PCM-DM is a useful tool for in vitro expansion of HCECs. Although CECs are hexagonal and monolayer tissues in vivo (Fig. 5A), they easily change to a fibroblastic phenotype and lose their hexagonal morphology and contact inhibition [20]. Thus, we tested whether PCM-DM was applicable to maintenance culture of HCECs. Interestingly, the cell density of the cultured HCECs on the PCM-DM was significantly higher than that of the control cells cultured on the noncoated dish (1803 cells/mm^2^ and 1231 cells/mm^2^, respectively). The HCECs cultured on the PCM-DM showed hexagonal and contact-inhibited hexagonal morphology similar to that observed in vivo, whereas the control HCECs showed a fibroblastic phenotype and loss of contact inhibition (Fig. 5B). As the density of CECs is a pivotal maker of healthiness in vivo, and the density is decreased when CECs are damaged by disease or senescence, we compared the effect of PCM-DM on the cell density to that of other ECMs. PCM-DM enhanced the cell density 1.7-fold, whereas collagen type 1, collagen type 4, and fibronectin doses did (Fig. 5C). Analysis of the endothelial characteristics of the two phenotypes revealed that the staining pattern of Na^+^/K^+^-ATPase and ZO-1 at the plasma membrane was well preserved in the HCECs cultured on PCM-DM, whereas this was disrupted in the control HCECs (Fig. 5D).

## Discussion

We demonstrated that PCM-DM provides a xeno-free culture substrate for efficient culture of HCECs. Several groups have reported HCEC cultivation techniques, and many researchers have attempted to develop viable HCEC culture protocols [18], [19], [34]. The pivotal technical obstacle when culturing HCECs is that they are vulnerable to morphological fibroblastic change under culture conditions, which disrupt the pump and barrier functions of the corneal endothelium [18], [20]. We recently demonstrated that activation of transforming growth factor-beta (TGF-β) signaling gives rise to massive fibroblastic change during culture conditions and that the use of SB431542, a selective inhibitor of the TGF-β receptor, counteracts the fibroblastic change and enables cultivation of HCECs, with the normal contact-inhibited monolayer associated with physiological functions [20].

Safety is another equally important issue to be addressed to enable clinical applications. Xenogenic materials need to be removed to avoid unexpected infections and acute rejection after transplantation [35]. We previously demonstrated that conditioned medium obtained from clinical-grade human bone marrow-derived mesenchymal stem cells instead of NIH-3T3 derived from mouse facilitated efficient cell proliferation of HCECs [36]. Another possible xeno-contamination source is the culture-supporting coating substrate. We propose that PCM-DM derived from human DMCs is a safe xeno-free substrate applicable to clinical use. A sufficient amount of DMCs (>10^9^ cells) can be routinely cultured after 4–5 weeks from 5×5 cm human decidua, and this amount of cells can provide PCM-DM for more than 3000 plates of a 3.5 cm culture dish. The DMCs may be freely available because they are derived from the maternal portion of human fetal adnexal tissues, which are otherwise discarded [27]. This availability makes PCM-DM a feasible substrate for CECs. Although GMP-grade gelatin was available for clinical use, we confirmed that DMCs exhibit high proliferating potency without gelatin to minimize the xeno-source. The finding that the inhibition of integrin α1, αv, and/or β1 subunits attenuates cell adhesion on the PCM-DM indicates that the effect of the PCM-DM on primate CECs is integrin dependent. It is well known that integrin-mediated environmental sensing plays an important role in the adaptation of the surrounding matrix by modulating cell proliferation, differentiation, and survival [33]. These cellular phenomena modulated by integrins are concordant with our findings that PCM-DM enhances cell adhesion, migration, proliferation, and survival. Although we have not identified the molecular components of PCM-DM that support efficient cultivation of CECs because of its complexity, PCM-DM is a complex of fibronectin, collagen IV, and other components [26]. The fact that defined substrates, such as collagen 1, collagen 4, and fibronectin, facilitate cell culture of HCECs but are less potent than PCM-DM suggests that PCM-DM as a human-derived material may be more useful for medical applications.

Further studies aimed at understanding how the cultivation of HCECs is augmented by PCM-DM will contribute to answering fundamental questions about why these cells exhibit less proliferative ability, even in vitro. The FBS supplemented in the culture medium is another possible xeno-source [37]. The use of certified prion-free FBS is not legally prohibited for tissue engineering, but it is necessary to develop a serum-free culture protocol.

Over 40,000 corneal transplantations were performed in 2011 in the United States [3], with corneal endothelial transplantation, such as DSAEK or DMEK, for treating corneal endothelial dysfunction representing over 40% of all corneal transplantations performed [38]. Favorable clinical results obtained with DSAEK or DMEK suggest that replacement of damaged corneal endothelium [3]–[5], [39], instead of full thickness replacement of the cornea, is sufficient to recover a transparent cornea. It should also encourage researchers to develop cultured corneal endothelial cell transplantation techniques as regenerative medicine. Several researchers, including ourselves, have transplanted cultured corneal endothelial sheets in animal models and showed that these are functional in vivo [8]–[12]. We recently reported that corneal endothelium can be regenerated without a carrier when cultured CECs are injected into the anterior chamber with Rho kinase inhibitor in rabbit and monkey corneal endothelial dysfunction models [13] because the Rho kinase inhibitor enhances the adhesion of CECs on the substrate [19]. In fact, clinical research in which cultured HCECs combined with Rho kinase inhibitor will be transplanted without a carrier was approved by the Ministry of Health, Welfare and Labor in Japan and is planned to be performed in the near future.

In conclusion, our current findings that PCM-DM enhances cell attachment, promotes cell proliferation, and suppresses apoptosis are applicable to clinical phase. The rapid developments taking place in tissue engineering techniques, combined with the establishment of a clinically applicable CEC culture protocol, will hopefully eventually offer patients a less invasive and highly effective therapeutic modality.
